# Reporting and Analyzing Race and Ethnicity in Orthopaedic Clinical Trials: A Systematic Review

**DOI:** 10.5435/JAAOSGlobal-D-21-00027

**Published:** 2021-05-21

**Authors:** Ryan W. Paul, Donghoon Lee, Joseph Brutico, Fotios P. Tjoumakaris, Michael G. Ciccotti, Kevin B. Freedman

**Affiliations:** From the Rothman Institute Orthopaedics, Thomas Jefferson University Hospital, Philadelphia, PA.

## Abstract

The distinction between race and ethnicity should be carefully understood and described for demographic data collection. Racial healthcare differences have been observed across many orthopaedic subspecialties. However, the frequency of reporting and analyzing race and ethnicity in orthopaedic clinical trials has not been determined. Therefore, the primary purpose of this systematic review was to determine how frequently race and ethnicity are reported and analyzed in orthopaedic clinical trials.

**Methods:**

The top 10 journals by impact factor in the field of orthopaedics were manually screened from 2015 to 2019. All randomized controlled trials related to orthopaedics and assessing clinical outcomes were included. Eligible studies were evaluated for bias using the Cochrane risk-of-bias tool and for whether the trial reported and analyzed several demographics, including age, sex, height, weight, race, and ethnicity. The frequency of reporting and analyzing by each demographic was accessed. In addition, comparisons of reporting and analyzing race/ethnicity were made based on orthopaedic subspecialty and journal of publication.

**Results:**

A total of 15,488 publications were screened and 482 met inclusion criteria. Of these 482 trials, 460 (95.4%) reported age and 456 (94.6%) reported sex, whereas 35 (7.3%) reported race and 15 (3.1%) reported ethnicity for the randomized groups; 79 studies (16.4%) analyzed age and 72 studies (14.9%) analyzed sex, whereas 6 studies (1.2%) analyzed race and 1 study (0.2%) analyzed ethnicity. The orthopaedic subspecialty of spine was found to report race (23.5%) and ethnicity (17.6%) more frequently than all the other subspecialties, whereas sports medicine reported race and/or ethnicity in only 3 of 150 trials (2.0%).

**Conclusions:**

Race and ethnicity are not frequently reported or analyzed in orthopaedic randomized controlled trials. Social context, personal challenges, and economic challenges should be considered while analyzing the effect of race and ethnicity on outcomes.

The distinction between race and ethnicity is complex, and a proper understanding of the differences between them is vital for demographic data collection.^1^ Race is a socially constructed term that associates individuals based on shared physical characteristics, whereas ethnicity is also a socially constructed term but instead categorizes people based on shared cultural identity and expression. In the United States, race categories often include American Indian or Alaska Native, Asian, Black, or African American, Native Hawaiian or Pacific Islander, and White or Caucasian, whereas ethnicity is defined as either Hispanic/Latino or not Hispanic/Latino.^2^ The National Institutes of Health guidelines require that minority patients be included in National Institutes of Health–funded research and that researchers collect patient-reported race and ethnicity.^3^ However, if previous research has shown that race and ethnicity do not affect the outcomes of an intervention, then race and ethnicity are not required as patient selection criteria, whereas their reporting and analysis are still strongly encouraged.^3^

Racial healthcare differences are driven heavily by the social determinants of health or factors outside of medicine that affect one's health, such as social norms, attitudes, and economic stability.^4^ These proximate factors may then lead to worse healthcare because of an inability to access care, a lack of health insurance, and provider biases. Racial health differences have been observed in several orthopaedic subspecialties, such as joint arthroplasty and spine surgery.^5^ In joint arthroplasty, Adelani et al^6^ controlled for comorbidities to more accurately evaluate the effects of race. They found that Black patients have an increased rate of complications and mortality after hip and knee arthroplasty, including postoperative infection, deep vein thrombosis, embolism, myocardial infarction, and stroke.^6^ Similar results have been observed in spine surgery, with Black patients having higher rates of mortality and complications after spine surgery than White patients.^7,8^ Because of these notable healthcare differences in patient populations among hospitals,^9^ orthopaedic research may benefit from frequent reporting of race and ethnicity demographics in publications.

According to CONSORT guidelines, randomized controlled trials (RCTs) should include all relevant demographic information in Table 1; ^10^however, what demographics are considered “relevant” is not clearly stated. Age and sex are almost always reported, whereas race and ethnicity are much less common. In a sample from four top medical journals, age and sex were reported in 99% of RCTs, whereas ethnicity was only reported in 37%.^11^ Similarly, Geller et al^12^ assessed the frequency of reporting race and ethnicity in prominent medical journals focusing on internal medicine, infectious disease, cardiology, oncology, and obstetrics and gynecology. They found that of 86 RCTs from 2009, 79% reported participants' race/ethnicity, whereas only 14% provided analysis by race/ethnicity.

**Table 1:** Reporting Rates of Age, Sex, Height, Weight, Race, and Ethnicity Among Seven Orthopaedic Surgery Subspecialties and One Nonsurgical Category

% of Papers Reporting Demographic	Foot/Ankle (n = 16)	Hand (n = 12)	Shoulder/Elbow (n = 12)	Spine (n = 17)	Sports (n = 150)	Total Joint (n = 137)	Trauma (n = 56)	Nonsurgical (n = 82)
Age	100.0	100.0	100.0	100.0	97.3	95.6	96.4	86.7
Sex	93.8	100.0	100.0	100.0	96.7	95.6	92.9	86.7
Height	31.3	0.0	16.7	11.8	14.0	19.7	7.1	22.9
Weight	37.5	0.0	25.0	23.5	26.7	44.5	7.1	28.9
Race	0.0	8.3	8.3	23.5	2.0	8.8	8.9	6.0
Ethnicity	0.0	0.0	0.0	17.6	0.7	3.6	1.8	6.0

The frequency of reporting race and ethnicity in orthopaedic RCTs has not been determined. Therefore, the primary purpose of this systematic review was to determine how frequently race and ethnicity are reported and analyzed in orthopaedic clinical trials. Our secondary purpose was to determine if reporting and analysis rates of race and ethnicity differ based on orthopaedic subspecialty and journal. Finally, our tertiary purpose was to evaluate how the included studies defined and analyzed both race and ethnicity.

## Methods

### Search Strategy and Criteria

This systematic review was done according to Preferred Reporting Items for Systematic Reviews and Meta-Analyses guidelines.^13^ Only journals related to orthopaedics, sports, bones, joints, and arthroscopy were considered. The journals with the 10 highest impact factors according to the 2019 Journal Citation Reports were selected. These journals were as follows: *British Journal of Sports Medicine*, *Bone Research*, *Sports Medicine*, *Journal of Bone and Mineral Research*, *American Journal of Sports Medicine*, *Journal of Sport and Health Science*, *Journal of Bone and Joint Surgery—American Volume*, *Clinical Orthopaedics and Related Research*, *Journal of Arthroscopy*, and *Bone & Joint Journal*. The publications from 2015 to 2019 were manually screened by two independent researchers (R.W.P. and D.L.) in September 2020, and any disagreements were settled by a third investigator (K.B.F.). A broad definition of orthopaedics was used, including orthopaedic surgery and topics related to injury prevention and exercise science.

### Inclusion and Exclusion Criteria

Studies were only included if a RCT study design was used, if a full-text publication was present, and if the topic was related to orthopaedics. Articles were excluded if they did not use a RCT design. Publications not related to orthopaedics were also excluded. Finally, studies that were basic science, a secondary analysis, or cluster randomized, were excluded.

### Assessment of Study Quality

Each article was evaluated for potential bias by the Cochrane risk-of-bias^14^ tool. The Cochrane risk-of-bias tool evaluates the bias of RCTs using seven categories: random sequence generation, allocation concealment, blinding of participants and personnel, blinding of outcome assessment, incomplete outcome data, selective reporting, and other biases. The risk of bias in each category was classified as high, low, or unclear.

### Data Collection and Abstraction

Age and sex served as frequently reported controls, whereas height and weight were used as infrequently reported controls. Full-texts were assessed to determine whether age, sex, height, weight, race, and ethnicity were reported for the study groups in each trial. Reporting a demographic variable was considered, providing a mean for continuous variables (age, height, and weight) or a sample size and/or percentage of the study population for categorical variables (sex, race, and ethnicity). Statistical analysis of demographics was also assessed as whether significant statistical analysis was done based on these variables relative to the study outcomes of interest. Comparing baseline demographics was not considered significant statistical analysis, whereas multivariate analyses or comparing subgroups divided based on one of these variables was considered significant analysis.

The following categories were used to classify subspecialty of orthopaedics: oncology, total joint and adult reconstructive surgery, spine, foot, and ankle, sports medicine, trauma, hand, and shoulder and elbow, and nonsurgical. The following race categories were used to guide our analysis: American Indian or Alaska Native, Asian, Black, or African American, Native Hawaiian or Pacific Islander, and White or Caucasian.^2^ Similarly, Hispanic and Latino were the considered ethnic categories of our review.^2^

The outcomes of interest were the frequency of reporting and analyzing race and ethnicity compared with other demographic variables (age, sex, height, and weight). Subspecialty category, whether the study reported and analyzed each demographic (0 = no and 1 = yes), and Cochrane Risk-of-Bias scores (0 = low, 1 = unclear, and 2 = high) were collected in Microsoft Excel. Assessments were completed among all orthopaedic RCTs and based on orthopaedic subspecialty.

## Results

**Figure 1:**
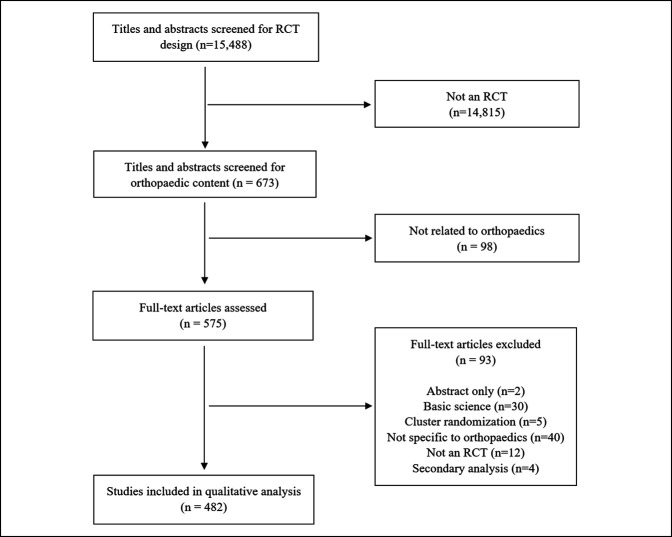
Flow chart showing the Preferred Reporting Items for Systematic Reviews and Meta-Analyses of study inclusion and exclusion, with 482 randomized controlled trials (RCTs) included in the qualitative analysis.

Details of paper inclusion/exclusion are presented in Figure 1. A total of 15,488 studies across 10 journals were screened for RCT design; 673 papers were then screened for relevance to orthopaedics. In total, 575 publications were fully assessed, and after excluding 93 studies for various reasons (Figure 1), 482 studies were included in the final qualitative analysis.

### Rates of Reporting Demographics

**Figure 2:**
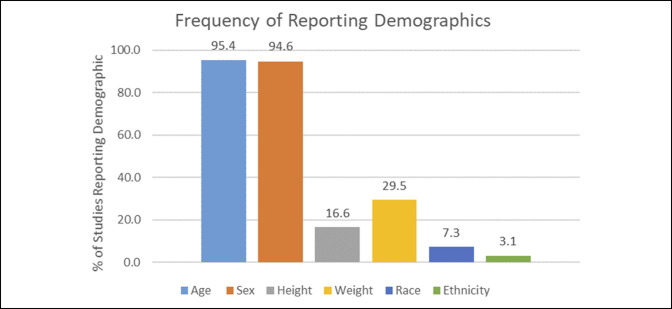
Bar diagram showing the percentage of orthopaedic randomized controlled trials that reported age, sex, height, weight, race, and ethnicity.

Of the 482 studies, 460 (95.4%) reported age and 456 (94.6%) reported sex for the randomized groups. Eighty (16.6%) reported height, and 142 (29.5%) reported weight for the randomized groups. Furthermore, 35 (7.3%) reported race, and 15 (3.1%) reported ethnicity for the randomized groups (Figure 2).

### Rates of Analyzing Demographics

**Figure 3:**
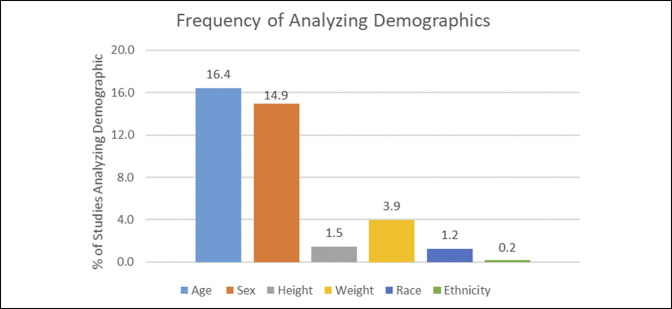
Bar diagram showing the percentage of orthopaedic randomized controlled trials that analyzed age, sex, height, weight, race, and ethnicity.

Of 482 studies, 79 studies (16.4%) analyzed age and 72 studies (14.9%) analyzed sex (Figure 3). Seven studies (1.5%) analyzed height, and 19 studies (3.9%) analyzed weight. Furthermore, six studies (1.2%) analyzed race, and only one study (0.2%) analyzed ethnicity (Figure 3).

### Included Studies that Reported and Analyzed Race and Ethnicity

Of the 35 RCTs that reported race, five studies^15,16,17,18,19^ only included one race (Table A1, http://links.lww.com/JG9/A133). The percentage of the participants that were White/Caucasian ranged from 29.9%^20^ to 98.3%,^21^ Black/African American ranged from 2.2%^22^ to 68.7%,^20^ Asian ranged from 0.8%^23^ to 60%,^24^ and Hispanic ranged from 0.9%^25^ to 39.6%^26^ (Table A1, http://links.lww.com/JG9/A133). In total, 20,686 patients in the 30 RCTs reported a study population of mixed race/ethnicity. Of these 20,686 patients, 130 (0.6%) were Asian, 1692 (8.2%) were Black/African American, 3590 (17.4%) were Hispanic, and 8933 (43.2%) were White/Caucasian.

### Orthopaedic Subspecialties

**Figure 4:**
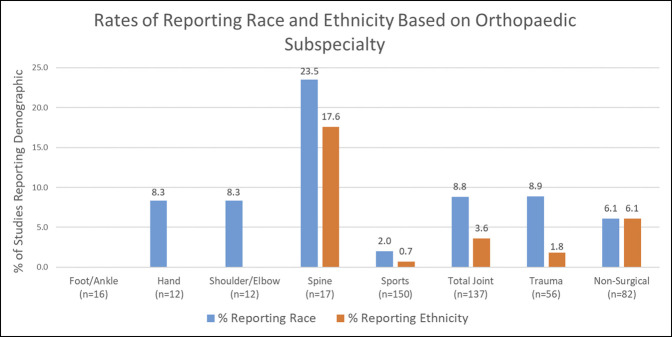
Bar diagram showing the rates of reporting race (blue) and ethnicity (orange) among seven orthopaedic surgery subspecialties and one nonsurgical category.

Sixteen publications were categorized as foot/ankle, 12 hand, 12 shoulder and elbow, 17 spine, 150 sports medicine, 137 total joint reconstruction and replacement, 56 trauma, and 82 nonsurgical. Spine reported both race (23.5%) and ethnicity (17.6%) more frequently than all other subspecialties (Table 1; Figure 4). Sports medicine, total joint reconstruction and replacement, and trauma reported race in 2.0%, 8.8%, and 8.9% of RCTs, respectively (Table 1; Figure 4). In addition, these subspecialties only reported ethnicity in 0.7%, 3.6%, and 1.8% of trials (Table 1; Figure 4). As for analysis, no spine and sports medicine RCTs analyzed race or ethnicity (Table 2). One total joint reconstruction and replacement RCT and two trauma RCTs analyzed race (Table 2). Finally, four nonsurgical RCTs analyzed race and one nonsurgical study analyzed ethnicity (Table 2).

**Table 2:** Analysis Rates of Age, Sex, Height, Weight, Race, and Ethnicity Among Seven Orthopaedic Surgery Subspecialties and One Nonsurgical Category

% of Papers Analyzing Demographic	Foot/Ankle (n = 16)	Hand (n = 12)	Shoulder/Elbow (n = 12)	Spine (n = 17)	Sports (n = 150)	Total Joint (n = 137)	Trauma (n = 56)	Nonsurgical (n = 82)
Age	6.3	25.0	16.7	17.6	12.0	15.3	30.4	16.9
Sex	6.3	25.0	8.3	23.5	11.3	11.7	17.9	24.1
Height	0.0	0.0	0.0	0.0	1.3	0.7	1.8	3.6
Weight	0.0	0.0	0.0	11.8	1.3	4.4	1.8	9.6
Race	0.0	0.0	0.0	0.0	0.0	0.7	1.8	4.9
Ethnicity	0.0	0.0	0.0	0.0	0.0	0.0	0.0	1.2

### Orthopaedic Journals

All journals reported age and sex more frequently than height, weight, race, and ethnicity (Table 3). Of all the journals, *Sport and Health* reported height and weight the most frequently (both 50.0%), whereas *Journal of Bone and Mineral Research* reported height least frequently (8.3%) and *American Journal of Sports Medicine* reported weight least frequently (23.6%). *Journal of Bone and Mineral Research* reported race and ethnicity most frequently (both 16.7%) (Table 3). *Arthroscopy* and *British Journal of Sports Medicine* did not report either race or ethnicity in any RCTs (Table 3). Analysis rates of these six demographics based on journal are also available in Table A2 (http://links.lww.com/JG9/A134).

**Table 3:** Reporting Rates of Demographics Based on Orthopaedic Journal

Journal	Reporting Age (%)	Reporting Sex (%)	Reporting Height (%)	Reporting Weight (%)	Reporting Race (%)	Reporting Ethnicity (%)
*American Journal of Sports Medicine* (n = 106)	98.1	99.1	13.2	23.6	6.6	2.8
*Arthroscopy* (n = 60)	93.3	91.7	13.3	25.0	0.0	0.0
*Bone & Joint Journal*(n = 94)	94.7	92.6	14.9	31.9	4.3	0.0
*British Journal of Sports Medicine* (n = 29)	96.6	96.6	37.9	37.9	0.0	0.0
*Bone Research* (n = 2)	100.0	100.0	50.0	0.0	0.0	0.0
*Clinical Orthopaedics and Related Research* (n = 51)	98.0	98.0	19.6	31.4	11.8	3.9
*Journal of Bone and Joint Surgery—American Volume* (n = 112)	94.6	94.6	11.6	29.5	11.6	7.1
*Journal of Bone and Mineral Research* (n = 12)	100.0	100.0	8.3	25.0	16.7	16.7
*Sport and Health* (n = 12)	75.0	58.3	50.0	50.0	25.0	0.0
*Sports Medicine* (n = 4)	100.0	100.0	50.0	75.0	0.0	0.0

### Cochrane Risk-of-Bias

**Figure 5:**
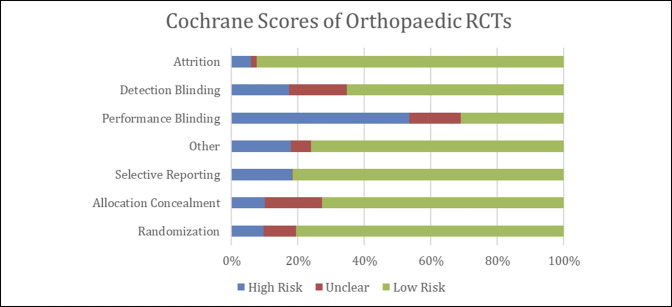
Diagram showing the Cochrane Risk-of-Bias scores of all included randomized controlled trials (RCTs). Green = low risk of bias, red = unclear, blue = high risk of bias.

The least amount of bias was present in the attrition category of the Cochrane risk-of-bias tool, with 92% of RCTs describing exclusion criteria and their participant dropout rates (Figure 5). The highest amount of bias was in the performance blinding category, with 53% of RCTs not blinding patients and intervention providers (Figure 5).

## Discussion

The primary purpose of our study was to evaluate the frequency of both reporting and analyzing race and ethnicity in orthopaedic RCTs. Race and ethnicity were rarely reported in orthopaedic RCTs, with only 7.3% of publications documenting race and 3.1% of publications documenting ethnicity. Race and ethnicity were also rarely analyzed, with only 1.2% of papers analyzing race and 0.2% of papers analyzing ethnicity. By evaluating orthopaedic subspecialties, we also found that the orthopaedic subspecialty of spine reported race and ethnicity more frequently than all the other subspecialties, whereas sports medicine reported race and ethnicity in only 3 of 150 RCTs and foot/ankle did not report either race or ethnicity in any of the included studies. Finally, by evaluating the reported demographics of included studies, we found that the racial and ethnic demographics of included patients ranged tremendously, and only two of six studies that analyzed race/ethnicity found differences based on race/ethnicity.

Of the six studies^20,27,28,29,30,31^ that analyzed race/ethnicity, two^30,31^ showed differences based on race/ethnicity. Warwick et al^30^ found through a logistic regression multivariable analysis that White patients were slightly more likely to respond to patient-reported outcome surveys compared with Black patients (odds ratio of 2.03) after orthopaedic surgery. Johnson et al^31^ calculated hazard ratios that found that African-American patients with type 2 diabetes mellitus may be at an increased long-term risk of fracture because of an intentional weight loss intervention (hazard ratio of 1.64), whereas White and Hispanic patients with type 2 diabetes mellitus are not (hazard ratios of 0.97 and 0.87, respectively). Interestingly, it should be noted that even with an intentional weight loss intervention, African-American patients still had a lower incidence of fractures (9.75%) than White patients (15.92%) and Hispanic patients (10.88%).^31^ However, racial and ethnic differences should not automatically be considered disparities.^32^ According to Rathore and Krumholz,^32^ a racial difference should only be considered a racial disparity if the difference cannot be explained by other patient factors. If a racial difference is shown not to be due to eligibility, clinical exclusion (contraindications), patient preferences, or confounding variables (demographic, clinical, and social) and the racial difference is associated with poorer patient outcomes, then the difference should be deemed a disparity.^32^

It is important to report and identify racial and ethnic differences throughout orthopaedics so that further examination can identify its root cause. For example, racial and ethnic differences have been clearly documented in total joint arthroplasty.^33,34^ Minority patients have been shown to have worse postoperative function and outcomes,^35^ and increased mortality rates.^36^ Despite these differences and their persistence throughout the years,^36^ RCTs have failed to frequently report these demographics, with RCTs in the total joint arthroplasty subspecialty reporting race and ethnicity in 8.8% and 3.6% of publications, respectively. Several proximal factors may contribute to these low rates, such as a lack of race/ethnicity data collection, a belief that reporting race/ethnicity is not clinically relevant, or a lack of emphasis to report race/ethnicity by medical journals. Educating researchers about the value of reporting and analyzing race and ethnicity, regardless of whether their studies identify or deny racial differences, may help improve reporting and analysis rates. In addition, medical journals may emphasize the reporting and analysis of race and ethnicity for prospective publications.

The complexity of interpreting findings regarding racial/ethnic differences may also lead to decreased reporting and analysis rates. It has been debated whether race and ethnicity are meaningful demographics in medical research.^37-39^ Race and ethnicity capture a lifetime of social experiences that may never be adequately controlled for^40,41^; thus, the effect of biological versus social factors cannot competently be discerned. According to a viewpoint by Cooper et al,^37^ medicine faces the challenge of needing to collect patient subgroup data to recognize and reduce inequities, while avoiding the misinterpretation and exaggeration of the effect race has on health. To combat this, clinicians and researchers should improve their understandings of social context and personal/economic challenges and should consider how social forces may have affected their data.^37^ Ultimately, research literature should become more effective at identifying differences and disparities, and clinicians may more effectively counsel patients before and after treatment.

This study is not without limitations. First, only RCTs related to orthopaedics were included. Studies regarding racial and ethnic differences are often cohort studies with a large sample size, but we did not include cohort studies. Second, no comparisons were made with fields outside of orthopaedics. It would be beneficial for future research to compare orthopaedics with other fields of medicine regarding rates of reporting and analyzing race/ethnicity and relate these findings to relevant social forces. Third, we did not evaluate other socioeconomic factors. Without evaluating additional social factors, an adequate evaluation of whether race/ethnicity should be reported and analyzed more frequently is not possible. Fourth, several subspecialties of orthopaedics were not included adequately. Foot and ankle, hand, shoulder and elbow, and spine surgery all had less than 18 RCTs included in this systematic review. Finally, this review did not isolate topics with notable health differences. It is possible that the included studies evaluated interventions where racial/ethnic differences are nonexistent or minimal, so reporting and analyzing race and/or ethnicity is not necessary.

In conclusion, race and ethnicity are not frequently reported or analyzed in orthopaedic RCTs. Orthopaedic spine surgery reported and analyzed race the most frequently of orthopaedic subspecialties, whereas all other orthopaedic subspecialties rarely reported race and ethnicity. Racial and ethnic demographics varied markedly among the RCTs that reported patient race and ethnicity. Social context, personal challenges, and economic challenges should be carefully considered while analyzing the effect of race and ethnicity on outcomes.
